# A Novel Electroosmotic Micromixer with Asymmetric Lateral Structures and DC Electrode Arrays

**DOI:** 10.3390/mi8040105

**Published:** 2017-03-29

**Authors:** Limin Chen, Yongbo Deng, Teng Zhou, Hui Pan, Zhenyu Liu

**Affiliations:** 1 Changchun Institute of Optics, Fine Mechanics and Physics (CIOMP), Chinese Academy of Science, Changchun 130033, Jilin, China; climber1990@126.com (L.C.); dengyb@ciomp.ac.cn (Y.D.); panhui13@mails.ucas.ac.cn (H.P.); 2 University of Chinese Academy of Sciences, Beijing 100049, China; 3 Mechanical and Electrical Engineering College, Hainan University, Haikou 570228, Hainan, China; zhouteng@hainu.edu.cn

**Keywords:** electroosmotic, micromixer, asymmetric electrode, lateral structure, mixing performance

## Abstract

We present a novel electroosmotic micromixer that consists of arrays of direct current (DC) asymmetric electrode and asymmetric lateral structures. By embedding asymmetric electrode arrays on the top and bottom walls of a rectangular microchannel appropriately, the flow perturbations and vortexes can be induced when a DC electric field is imposed. An efficient lateral structure is then sequentially combined with the rectangular microchannel, which enhances the mixing effect significantly. The effects of operational parameters such as the Reynolds number, the applied potential, and the Peclet number on the mixing performance are analyzed in detail by numerical simulations. The results indicate that an enhanced mixing performance can be achieved with low applied potential. The novel method proposed in this paper provides a simple solution for mixing in the field of micro-total-analysis systems.

## 1. Introduction

Rapid and complete mixing of two or more reagents in many microfluidic systems such as lab-on-a-chip (LOC) is key. However, in both pressure- and electrokinetically driven flow microfluidic systems, the Reynolds number is inherently limited to the laminar regime. Consequently, the flow is lacking turbulence, and the mixing of the reagents is dominated by molecular diffusion effects. Such an approach tends to require a long mixing time and mixing distance to achieve complete mixing performance. Therefore, micromixers with efficient mixing performance are strongly desired in a microfluidic system, a continuously studied topic [1,2].

Many novel micromixers have been developed in recent years. Based on actuation methods, these micromixers can be classified into passive micromixers and active micromixers [3,4,5]. Passive micromixers enhance mixing by using specially designed channel geometry or adding geometric obstacles, and the mixing process relies entirely on diffusion or chaotic advection mechanisms [6,7,8,9]. Active micromixers, on the other hand, achieve mixing by using types of external energy, such as pressure [10], temperature [11], magnetohydrodynamic [12,13,14], acoustics/ultrasonic [15,16], and electrokinetic [17,18,19,20,21,22]. Compared to passive mixing, active mixing generally has a shorter mixing time and mixing distance and allows users to turn on and off the mixing enhancement if necessary [23,24]. Among all the mentioned active actuations, electroosmotic flow (EOF) is one of the most widely used to enhance the mixing effect [25,26]. Electrokinetic mixers can also be divided into active electrokinetic mixers and passive electrokinetic mixers. Passive electrokinetic mixers enhance mixing by their surface characteristics, geometry shapes, and lack of stability under a direct current (DC) voltage with active electrokinetic mixers by using a time-dependent electric field or through an externally time-dependent or -independent electrical force [27,28,29,30,31,32,33,34,35].

Topology optimization has been widely applied in the design of microfluidic devices because inexperienced designers can obtain a better structural topology according to design goal [36,37,38,39,40,41,42,43,44]. Topology optimization of the fluid was first applied by Borrvall and Petersson to the Stokes flow [45], and later to Navier–Stokes flow [46,47,48,49,50,51]. By distributing electrodes on both sides of the microchannel, Deng et al. obtained the optimal distribution of the electrodes based on topology optimization method [43], which shows it is an effective active mixing approach. In order to obtain a more efficient performance using this active mixing approach, one can increase the electrode voltage or increase the number of electrode. Increasing the voltage may cause undesired effects like heat or bubble generation and change of fluid properties such as pH. Increasing the number of electrodes increases the manufacturing cost and complexity, and the longer length of channel might cause the miniaturization problem of microfluidic devices. Effective passive mixers such as the 3D serpentine micromixer [52] or the staggered herringbone micromixer [53] generally have a complex 3D structure. However, it is a challenge to fabricate complex 3D geometries. Based on the Dean effect, Zhou et al. designed a single layer passive mixer with an optimized lateral structure via the topology optimization method [39]. The mixer is easier to manufacture for a single layer design. However, due to the passive mixing principle, the lateral structure is designed larger than the microchannel in order to allow the fluid flow into the lateral structure.

**Figure 1:**
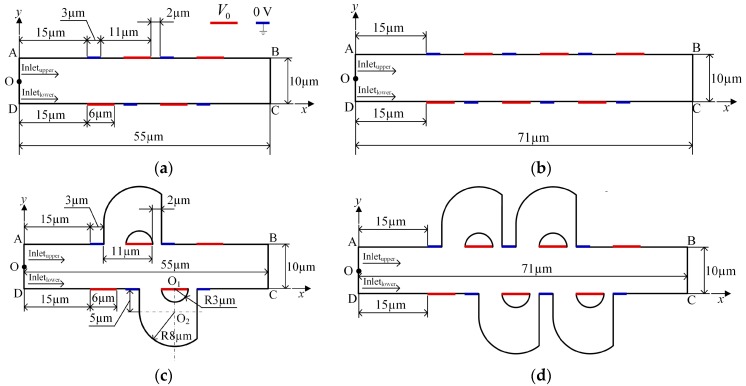
Schematic illustrations of micromixers with (**a**) 8 asymmetric electrodes (8E), (**b**) 12 asymmetric electrodes (12E), (**c**) 8 asymmetric electrodes and two asymmetric lateral structures (8E2L), and (**d**) 12 asymmetric electrodes and four asymmetric lateral structures (12E4L). AD: inlet, BC: outlet, O: midpoint of boundary AD.

In order to overcome the shortcomings mentioned above, we present here a rapid EOF mixer with more compact asymmetric lateral structures and DC asymmetric electrode arrays. The compact lateral structure was obtained based on the topography optimization result by [38,39]. Due to the compact design, it presents negligible influence on the switch ability of the active mixer. The distribution of the electrodes arrays was adjusted according to the work of [43]. In order to compare the mixing performance, we also compared the mixer without lateral structure, as shown in Figure 1. Numerical simulations were conducted to illuminate the efficiency of the proposed EOF micromixer with lateral structures, and the mixing performance is significnatly improved compared with the EOF micromixer with DC asymmetric electrode arrays alone. Since the electric field is stationary, the driving component of the proposed mixer is simple and suitable for connection with other microfluidic functional components.

## 2. The Mathematical Model and Numerical Method

In this study, two-dimensional (2D), steady-state, incompressible, and viscous flow in a slit-type microchannel with asymmetric lateral structures and DC asymmetric electrode arrays is analyzed, as shown in Figure 1. The channel aspect ratio is supposed to be large enough for the 2D simulation analysis to be relevant. Period distribution microelectrodes are positioned on the side walls of the microchannel, and asymmetric lateral structures that connect the two sides of the long electrode are added to the microchannel. Figure 1a,b show that the rectangular channel periodically distributed long and short electrodes on the top and bottom walls, and the distance between adjacent electrodes is 5 μm and 2 μm, respectively. The electrodes on the side walls have an interlaced arrangement. Figure 1a shows the mixer with 8 electrodes, and Figure 1b with 12 electrodes; Figure 1c is obtained by adding two asymmetrical lateral structures to Figure 1a, and Figure 1d is obtained by adding four lateral structures to Figure 1b.

### 2.1. Mathematical Model

The fluid in the microchannel is Newtonian and incompressible. Since the thickness of the electrical double layer (EDL) is very thin compared to the width of the microchannel, the thin EDL approximation is valid for microscale electrokinetics involved in the present study. Therefore, the net charge density in the computational domain is zero and the electric potential *φ* satisfies the Laplace equation,

(1) $$\nabla^{2}\phi =0\;\begin{array}{c} in\;\Omega \end{array}$$

The boundary conditions for *φ* on the asymmetric electrode arrays are

(2) $$\phi =\phi_{0}\;\mathrm{on}\;\mathrm{the}\;\mathrm{long}\;\mathrm{electrodes}$$

and

(3) ϕ=0 on the short electrodes

Other boundaries (including the channel wall, except on electrodes), inlets, and outlets are electrically insulating,

(4) n⋅∇ϕ=0 on other boundaries

The numerical results presented for the micromixers are based on the incompressible Navier–Stokes equations. The conservation of mass and momentum in the fluid are thus given by

(5) ∇⋅u=0 in Ω

and

(6) $$\rho (u\cdot \nabla )u=-\nabla p+\eta \nabla^{2}u\;in\;\Omega$$

where **u** is the fluid velocity vector, *p* is the pressure, and *ρ* and *η* are, respectively, the fluid density and the dynamic viscosity. Because of its low Reynolds number, the flow field in the mixing channel is supposed to be laminar and exhibits a parabolic profile with a mean flow velocity *U*_0_ at two inlets. The fluid velocity adjacent to the microchannel wall is approximated by the Smoluckowski slip velocity,

(7) $$u=\frac{\varepsilon_{0}\varepsilon_{f}\zeta_{w}}{\eta }(I-\mathrm{nn})\cdot \nabla \phi \;\mathrm{on}\;\mathrm{charged}\;\mathrm{channel}\;\mathrm{wall}$$

where $\varepsilon_{0}$ and $\varepsilon_{f}$ are, respectively, the permittivity of vacuum and the relative permittivity of fluid. $\zeta_{w}$ is the zeta potential of the channel wall. **I** and **n** are, respectively, the second-order unit tensor and the unit normal vector pointing from the channel wall to the fluid domain.

The ambient pressure and no-traction condition are applied at outlet BC:

(8) $$p=0\;\mathrm{on}\;\Gamma_{\mathrm{BC}}$$

(9) $$\eta \nabla u\cdot n=0\;\mathrm{on}\;\Gamma_{\mathrm{BC}}$$

In addition, the following convection–diffusion equation is for the concentration *c* of the dissolved substances in the fluid:

(10) u⋅∇c=∇⋅(D∇c) in Ω

where *c* and *D* are, respectively, the concentration and the diffusion coefficient. For the steady convection–diffusion equation, the concentrations at inlets AO and OD are specified as

(11) $$c=0\;mol/m^{3}\;on\;\Gamma_{\mathrm{AO}}$$

(12) $$c=1\;mol/m^{3}\;on\;\Gamma_{\mathrm{OD}}$$

while the condition of no species flux is imposed at channel walls,

(13) (cu−D∇c)⋅n=0  on channel wall

The boundary condition along the outlet is

(14) $$(D\nabla c)\cdot n=0\;\mathrm{on}\;\Gamma_{\mathrm{BC}}$$

The Reynolds number is defined as

(15) Re=ρU0Wη

where W=10 μm is the width of the microchannel. The Peclet number is defined as

(16) $$\mathrm{Pe}=\frac{U_{0}W}{D}$$

### 2.2. Numerical Method

The commercial finite element package COMSOL Multiphysics (Version 5.2a, COMSOL Group, Stockholm, Sweden) is used to perform the numerical simulation. The density of the fluid is 1 × 10^3^ kg/m^3^, and the dynamic viscosity is 1 × 10^−3^ Pa·s. The relative permittivity of fluid is 80.2 and the permittivity of vacuum is 8.854 × 10^−12^ F/m. The zeta potential of the channel wall equal to −0.1 V. The least-square type optimization objective can be used to express the mixing effect of the two flows with an anticipated distribution of the concentration near the outlet as [24,37,38,39], which is used as the mixing performance index,

(17) $$\sigma =\sqrt{\frac{\int_{\Gamma }{\left(c-c_{ideal}\right)}^{2}}{Lc_{ideal}^{2}}}$$

where $c_{ideal}=0.5$ corresponds with perfect mixing on a normalized scale, c is the concentration distribution at outlet Γ_BC_, and *L* is the length of the outlet. Complete segregation and complete mixing are then defined by σ=1 and σ=0, respectively. Therefore, the higher the value is, the more inefficient the performance of the mixer is.

## 3. Results and Discussion

In this section, the concentration distribution, the electric potential surface plot and fluid streamlines (magnitude controlled positioning) are used to present the mixing mechanism and performance of the micromixers. Furthermore, the mixing performance index *σ* of the mixers versus the potential, the Reynolds number (the inlet mean velocity), and the Peclet number (the diffusion coefficient) is analyzed to show the influence of these three parameters.

### 3.1. Mixing Mechanism

**Figure 2:**
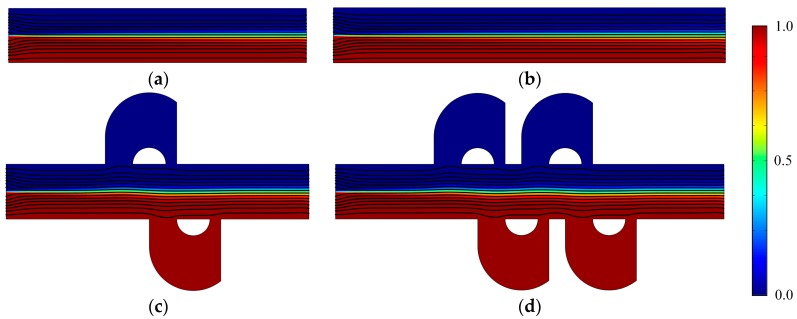
Fluid streamlines and the concentration distribution in the channel of (**a**) the mixer with 8E, (**b**) the mixer with 12E, (**c**) the mixer with 8E2L, and (**d**) the mixer with 12E4L in the absence of an electric field, and the mean inlet velocity *U*_0_ = 1 × 10^−3^ m/s (Re = 1 × 10^−2^). The unit for the concentration is mol/m^3^.

Figure 2 shows the concentration distribution and fluid streamlines of the four mixers without an electric field applied. The mean inlet velocity and the diffusion coefficient are *U*_0_ = 1 × 10^−3^ m/s and *D* = 1 × 10^−11^ m^2^/s, respectively. From the results shown in Figure 2, we can see that inefficient mixing performance is presented in the microflows with a Reynolds number of 1 × 10^−2^ and a Peclet number of 1 × 10^3^, due to the absence of an electric field. Although the mixers (Figure 2c,d) have two more and four more lateral structures, respectively, than straight channel mixers (Figure 2a,b), the inlets of the lateral structure are relatively smaller than the width of the channel and the flow rate in the lateral structure is small. In addition, even though there is fluid flowing into the lateral structure, it always flows into the closer one in which there is no mixing process. From the fluid streamlines, one can clearly see that the fluid flows nearly along the direction of the pipeline with little perturbation, which can be almost negligible in the vicinity of the lateral structures. In general, there is nearly no improvement of the mixing effect in the mixers with the lateral structures for the case without an electric field applied. This means the mixers with lateral structures retain the switching characteristics of the active mixers, namely, when there is no need to mix, just turn off the voltage.

**Figure 3:**
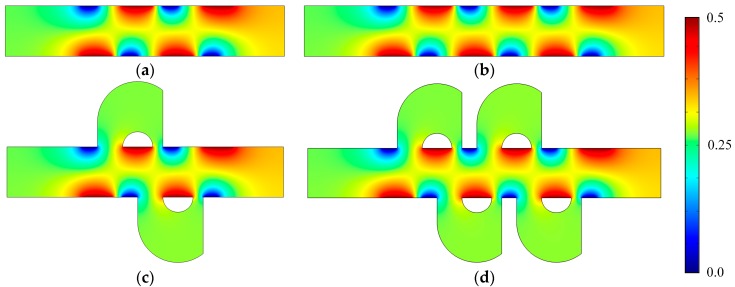
Electric potential surface plot of (**a**) the mixer with 8E, (**b**) the mixer with 12E, (**c**) the mixer with 8E2L, and (**d**) the mixer with 12E4L when the device uses the potentials *V*_0_ = 0.5 V and the inlet mean velocity *U*_0_ = 1 × 10^−3^ m/s (Re = 1 × 10^−2^).

**Figure 4:**
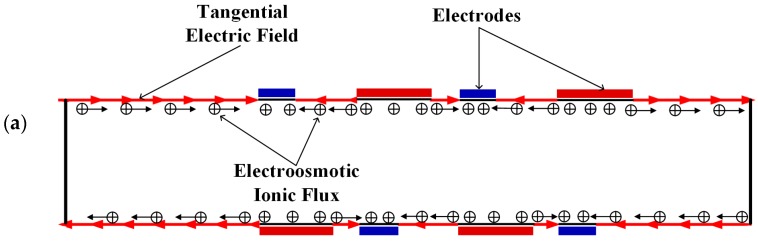

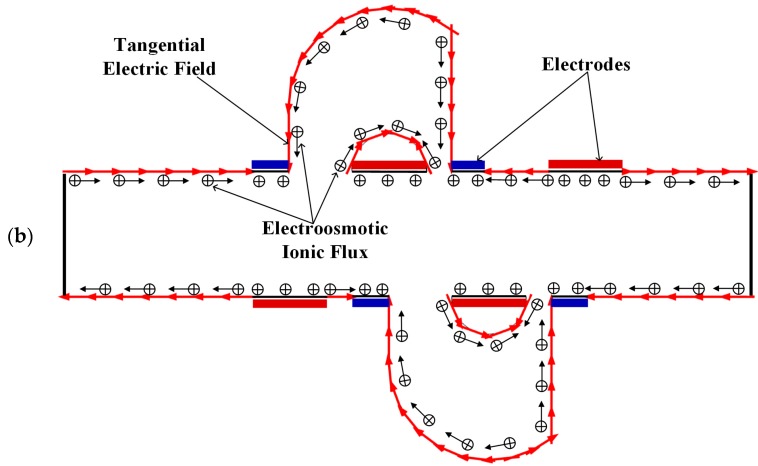
Schematic of the generation of the electroosmotic flow in (**a**) the mixer with 8E and (**b**) the mixer with 8E2L.

**Figure 5:**
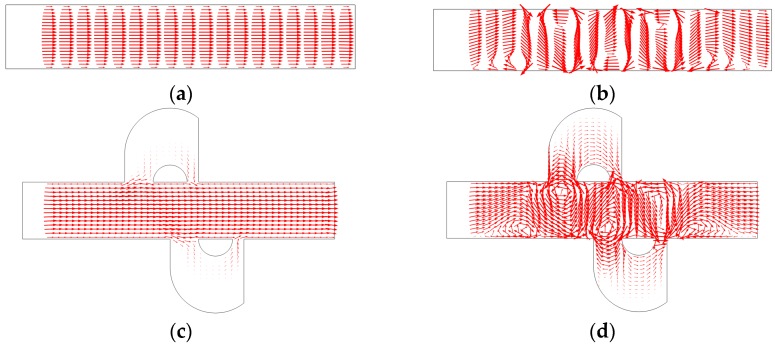
The velocity distribution of (**a**) the mixer with 8E without an electric field applied, (**b**) the mixer with 8E with an electric field applied, (**c**) the mixer with 8E2L without an electric field applied, and (**d**) the mixer with 8E2L with an electric field applied when the device uses the potentials *V*_0_ = 0.5 V and the inlet mean velocity *U*_0_ = 1 × 10^−3^ m/s (Re = 1 × 10^−2^).

**Figure 6:**
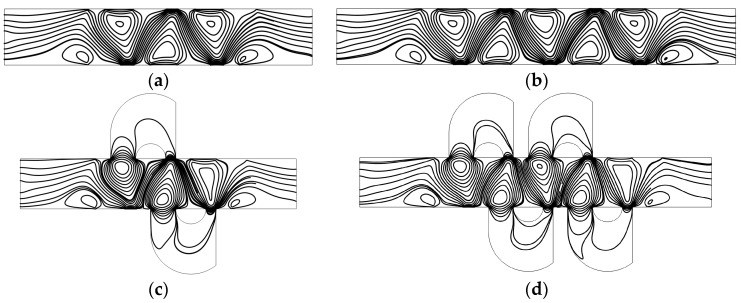
Fluid streamlines in the channel of (**a**) the mixer with 8E, (**b**) the mixer with 12E, (**c**) the mixer with 8E2L, and (**d**) the mixer with 12E4L when the device uses the potentials *V*_0_ = 0.5 V and the inlet mean velocity *U*_0_ = 1 × 10^−3^ m/s (Re = 1 × 10^−2^).

**Figure 7:**
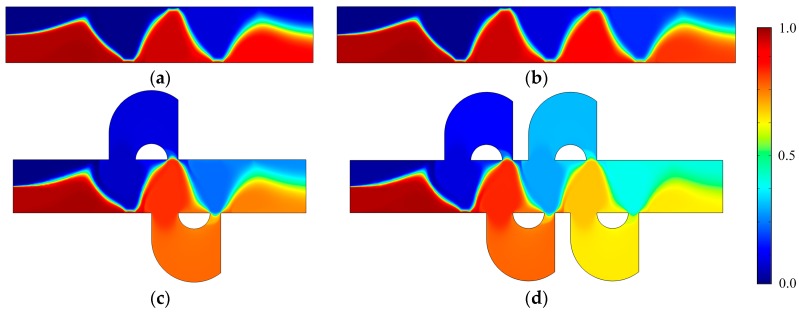
The concentration distribution of (**a**) the mixer with 8E, (**b**) the mixer with 12E, (**c**) the mixer with 8E2L, and (**d**) the mixer with 12E4L when the device uses the potentials *V*_0_ = 0.5 V and the inlet mean velocity *U*_0_ = 1 × 10^−3^ m/s (Re = 1 × 10^−2^). The unit for the concentration is $mol/m^{3}$.

Figure 3 shows the electric potential surface plot of the four mixers with an electric field applied. The applied voltage for all mixers is *V*_0_ = 0.5 V. In Figure 3, one can see that there is a large electric potential gradient between adjacent electrodes, and the high electric potential gradient results in a large electric force acts on the fluid. On the other hand, the interlaced arrangement of the electrodes on the side walls avoid the counteraction of the electric force effectively. Figure 4 shows how the electric field force acts on the fluid. The negative zeta potential implies a negative fixed surface charge density on the channel walls and thereby positive counterions within the EDL. The interaction of positive counterionic charge fixed in the EDL (merely related to the surface charge) with the applied tangential field (normal component is insulated by the EDL capacitance, in accordance with the thin double layer approximation used here) results in electroosmotic slip flow in the direction of the local electric field. As shown in Figure 4, due to the horizontal staggered distribution, and the longitudinal one-to-one distribution of the long electrodes and short electrodes, the direction of electroosmotic flow on both sides of the electrode is opposite, and the direction of electroosmotic flow on the two opposite channel walls is opposite. First, this will cause rotating vortices, which increase the disturbance and the chaotic advection. Second, this will force the fluid zigzag to flow in a narrow area, which increases the contact distance and the local concentration gradients between the fluids, leading to a greater diffusive flux and inducing an efficient mixing. Figure 5 shows the velocity distribution of the fluid field in the mixer with 8E and mixer with 8E2L without an electric field applied and with an electric field applied, respectively. The mixers (Figure 3c,d) simply connect the insulated walls on both sides of the long electrodes by lateral structures, this will not influence the electric field distribution in the channels, the fluid can be sufficiently disturbed by the generated vortexes and the distortion of the streamline. In Figure 6c,d, we can also found that, due to the existence of lateral structures, the vortexes in the channels are induced more effectively, and the vortexes are enlarged by the lateral structures, which enhance the chaotic advection significantly. At the same time, there is more fluid flowing into the lateral structures for mixing, increasing the effective mixing area. Finally, Figure 7 shows the concentration distribution of the four mixers. It is worth noticing that the mixing effect is enhanced, which in turn indicates that the addition of two lateral structures performs more efficiently than the addition of four electrodes under the current voltage and Reynolds number. By adding the electrode and lateral structures simultaneously, the mixing effect can be markedly enhanced.

### 3.2. Mixing Performance

**Figure 8:**
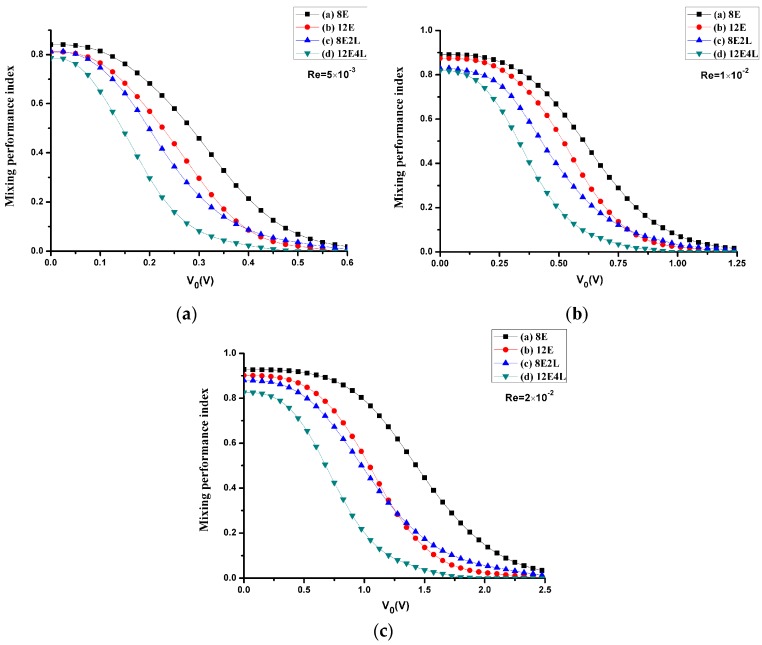
The mixing performance index *σ* versus the potential *V*_0_, where the Reynolds number is (**a**) 5 × 10^−3^, (**b**) 1 × 10^−2^, and (**c**) 2 × 10^−2^ (the corresponding inlet mean velocity *U*_0_ is (**a**) 5 × 10^−4^ m/s (**b**) 1 × 10^−3^ m/s (**c**) 2 × 10^−3^ m/s). Black line with square symbols: the mixer with 8E; red line with circular symbols: the mixer with 12E; blue line with regular triangle symbols: the mixer with 8E2L; green line with inverted triangle symbols: the mixer with 12E4L.

We calculate the mixing performance index σ of four mixers considering the Reynolds number and the electrical potential with the diffusion coefficient *D* = 1 × 10^−11^ m^2^/s. One can see in Figure 8 that the mixing performance of all mixers at three different Reynolds numbers is improved with the increase in the applied voltage, and the maximum voltage reached at complete mixing increases as the Reynolds number increases, which is because the electroosmotic velocity is proportional to the gradient of the voltage. In all cases, the most inefficient one is mixer (Figure 1a), and mixer (Figure 1d) most efficient, while mixer (Figure 1b) and mixer (Figure 1c) have similar performances. However, when the applied voltage is smaller, the performance of adding lateral structures is more efficient; the performance of mixer (Figure 1b) is enhanced compared with mixer (Figure 1c) when the voltage increases continuously, since the effect of adding four electrodes at higher voltages is better than that of adding two lateral structures, but the difference is not so significant. Therefore, we can conclude that the combination of the electrodes and lateral structures can effectively reduce the mixing voltage, while deriving a more efficient mixing performance compared with the cases that only adding electrodes or lateral structures.

**Figure 9:**
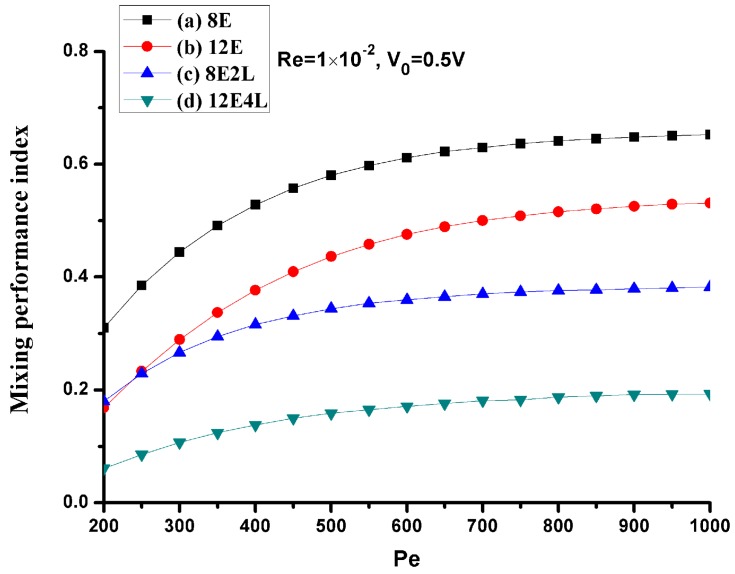
The mixing performance index *σ* versus the Peclet number for *U*_0_ = 1 × 10^−3^ m/s (Re = 1 × 10^−2^) and *V*_0_ = 0.5 V. The horizontal and vertical axes depict the Peclet number and mixing performance index *σ*, respectively. Black line with square symbols: the mixer with 8E; Red line with circular symbols: the mixer with 12E; Blue line with regular triangle symbols: the mixer with 8E2L; Green line with inverted triangle symbols: the mixer with 12E4L.

Since the Peclet number has a great effect on the mixing, we consider mixing under different Peclet numbers at a mean velocity of *U*_0_ = 1 × 10^−3^ m/s (Re = 1 × 10^−2^), the Peclet numbers range from 200 to 1000 (the corresponding diffusion coefficient *D* values range from 5 × 10^−11^ m^2^/s to 1 × 10^−11^ m^2^/s), as shown in Figure 9. When the Peclet number becomes smaller, the diffusion is stronger at this time, and the performance of all mixers is more efficient, but as the Peclet number increases, the performance of the mixers without lateral structures rapidly deteriorates, and mixers with lateral structures perform more efficiently. This shows that, when the Peclet number increases, the lateral structures will effectively enhance the convection.

## 4. Conclusions

This paper proposes an effective mixer with more compact asymmetric lateral structures and DC asymmetric electrodes, where the realization of the mixing is dependent on both the electroosmotic flow generated by the electric field and the enhanced convection by lateral structures. The mixing effect at different applied voltages, Reynolds numbers, and Peclet numbers was studied. The simulation results indicate that the mixing effect is more efficient in all cases where lateral structures are added because the lateral structures expand the induced vortexes and increase the effective mixing area. Due to the inlets of the lateral structures are smaller than the width of the channel, they present negligible influence on the switch ability of the active mixer. The lateral structures can be manufactured by standard photolithography, so it is possible to reduce the manufacturing cost. Future work will be done experimentally to validate the effect of the proposed mixer.
